# A *SEL1L* Mutation Links a Canine Progressive Early-Onset Cerebellar Ataxia to the Endoplasmic Reticulum–Associated Protein Degradation (ERAD) Machinery

**DOI:** 10.1371/journal.pgen.1002759

**Published:** 2012-06-14

**Authors:** Kaisa Kyöstilä, Sigitas Cizinauskas, Eija H. Seppälä, Esko Suhonen, Janis Jeserevics, Antti Sukura, Pernilla Syrjä, Hannes Lohi

**Affiliations:** 1 Department of Medical Genetics, University of Helsinki, Helsinki, Finland; 2 Department of Veterinary Biosciences, University of Helsinki, Helsinki, Finland; 3 Department of Molecular Genetics, Folkhälsan Institute of Genetics, Helsinki, Finland; 4 Referral Animal Neurology Hospital Aisti, Vantaa, Finland; 5 Small Animal Clinic Kontiolahti, Kontiolahti, Finland; University of Bern, Switzerland

## Abstract

Inherited ataxias are characterized by degeneration of the cerebellar structures, which results in progressive motor incoordination. Hereditary ataxias occur in many species, including humans and dogs. Several mutations have been found in humans, but the genetic background has remained elusive in dogs. The Finnish Hound suffers from an early-onset progressive cerebellar ataxia. We have performed clinical, pathological, and genetic studies to describe the disease phenotype and to identify its genetic cause. Neurological examinations on ten affected dogs revealed rapidly progressing generalized cerebellar ataxia, tremors, and failure to thrive. Clinical signs were present by the age of 3 months, and cerebellar shrinkage was detectable through MRI. Pathological and histological examinations indicated cerebellum-restricted neurodegeneration. Marked loss of Purkinje cells was detected in the cerebellar cortex with secondary changes in other cortical layers. A genome-wide association study in a cohort of 31 dogs mapped the ataxia gene to a 1.5 Mb locus on canine chromosome 8 (p_raw_ = 1.1×10^−7^, p_genome_ = 7.5×10^−4^). Sequencing of a functional candidate gene, sel-1 suppressor of lin-12-like (*SEL1L*), revealed a homozygous missense mutation, c.1972T>C; p.Ser658Pro, in a highly conserved protein domain. The mutation segregated fully in the recessive pedigree, and a 10% carrier frequency was indicated in a population cohort. SEL1L is a component of the endoplasmic reticulum (ER)–associated protein degradation (ERAD) machinery and has not been previously associated to inherited ataxias. Dysfunctional protein degradation is known to cause ER stress, and we found a significant increase in expression of nine ER stress responsive genes in the cerebellar cortex of affected dogs, supporting the pathogenicity of the mutation. Our study describes the first early-onset neurodegenerative ataxia mutation in dogs, establishes an ERAD–mediated neurodegenerative disease model, and proposes *SEL1L* as a new candidate gene in progressive childhood ataxias. Furthermore, our results have enabled the development of a genetic test for breeders.

## Author Summary

Hereditary ataxias are a heterogeneous group of rare disorders characterized by progressive cerebellar neurodegeneration. Several causative mutations have been identified in various forms of human ataxias. In addition to humans, inherited ataxias have been described in several other species, including the domestic dog. In this study, we have studied the clinical and genetic properties of cerebellar ataxia in the Finnish Hound dog breed. The breed suffers from a progressive ataxia that has an early onset before the age of 3 months. Affected puppies have difficulties in coordinating their movements and balance, and have to be euthanized due to rapidly worsening symptoms. Our pedigree analysis suggested an autosomal recessive mode of inheritance, which was confirmed by identifying a homozygous mutation in the *SEL1L* gene through genome-wide association and linkage analyses. The SEL1L protein functions in a protein quality control pathway that targets misfolded proteins to degradation in the endoplasmic reticulum. Mutations in the *SEL1L* gene have not been previously found in ataxias. Our study indicates *SEL1L* as a novel candidate gene for human childhood ataxias, establishes a large animal model to investigate mechanisms of cerebellar neurodegeneration, and enables carrier screening for breeding purposes.

## Introduction

Ataxia is a neurological symptom of defective motor coordination that can affect gait, balance, speech and gaze [1]. Human hereditary ataxias are rare heterogeneous disorders characterized by progressive degeneration of the cerebellum and cerebellar connections, with a variable degree of involvement from extra-cerebellar structures [2]. The predominant inheritance patterns are autosomal dominant and autosomal recessive [1]. Unlike the autosomal dominant spinocerebellar ataxias (SCAs), which usually affect the central nervous system (CNS), the recessive disorders involve more often other organs [1]. Typical age of onset for dominant ataxias is between 30 to 50 years of age [3], whereas the recessive forms tend to have an onset before the age of 20 years [4].

Causative mutations have been identified for at least 19 different dominant SCAs, most of which are caused by repeat expansions [5], [6]. In recessive human ataxias, the number of known disease genes is somewhere around 20, depending on the classification criteria [2], [7]–[10]. Described pathological mechanisms are diverse but include some common themes, such as accumulation of protein aggregates, defects in the DNA-repair system, mitochondrial dysfunction and oxidative stress [1], [2], [9], [11]. In addition to the known human ataxia genes, several spontaneous mutations that cause cerebellar degeneration have been recognized in mice [12]–[14].

Cerebellar degeneration has also been described in several dog breeds [15]–[30]. In veterinary medicine, the disease group is referred to as cerebellar cortical abiotrophies (CCAs), where abiotrophy describes the idiopathic premature neuronal degeneration [31]. Clinical signs in canine CCAs include ataxia, dysmetria, tremors, broad-based stance and loss of balance, all of which contribute to the often significant ambulatory difficulties [32], [33]. Majority of the described canine phenotypes are early-onset and manifest by the age of 3 to 4 months [16], [17], [19]–[23], [25], [27], [28]. Later-onset and slowly progressing CCAs are less common but occur in some breeds [18], [24], [26]. In a classical CCA, pathological findings are focused on the cerebellar cortex where the primary degenerative change is the loss of cortical Purkinje cells (PCs), followed by secondary changes in granular and molecular cell layers [32], [33]. Primary degeneration of cortical granule cells is seen more rarely [27], [29]. Involvement of CNS structures other than the cerebellum has been reported in some breeds, for instance in Kerry Blue Terriers [16] and Brittany Spaniels [24]. A more systemic phenotype is seen in the Bernese Mountain Dog, where cerebellar degeneration is accompanied by a hepatic degeneration [23]. In Rhodesian Ridgebacks, affected dogs present with a diluted coat color [22]. Collectively, the variability in disease onset, severity and histopathological details indicate a heterogeneous genetic etiology across different breeds. Although autosomal recessive inheritance has been proposed in several breeds [16], [18], [26], [30], the underlying genetic causes of canine primary ataxias have remained largely unidentified. Thus far, a molecular characterization has been reported only in a rare type of neonatal ataxia in Coton De Tulear dogs that have a mutation in the *GRM1* glutamate receptor gene [34]. Additionally, a putative CCA locus has been recently mapped to canine chromosome 3 (CFA3) in Australian Kelpies [35].

In the present study, we have examined clinical and genetic characteristics of hereditary ataxia that affects the Finnish Hound (FH) dog breed. A previous case report has indicated an early-onset progressive cerebellar neurodegeneration in a FH puppy [15]. We provide a more comprehensive clinical picture in a larger sample cohort and identify a recessive mutation in a novel ataxia gene.

## Results

### Clinical examinations indicate generalized cerebellar ataxia

Ten affected FH puppies from six different litters were referred to a veterinary neurology clinic for general clinical, orthopedical and neurological examinations (Table 1). One healthy littermate was examined as a control dog. At the time of examination, affected puppies were from 3 to 4 months old. The clinical signs were first noticed at a mean age of 9 weeks, ranging from 4 to 12 weeks (Table 1). General clinical and orthopedical examination did not reveal any significant changes. Neurological examinations were strongly indicative of cerebellar dysfunction by revealing generalized cerebellar ataxia with dysmetria (Video S1), postural reaction deficits (Video S2) and intention tremor (Video S3). Cranial and spinal nerve reflexes were normal, and all affected dogs had normal cognition. Serum biochemistry profiles, complete blood cell count (CBC) and cerebrospinal fluid (CSF) cell count were within normal limits in all affected dogs. For an undefined reason, protein concentration was mildly elevated in one affected puppy. All ten affected puppies were euthanized because of a rapid disease progression and poor prognosis.

**Table 1:** A summary of clinical and pathological examinations in Finnish Hound puppies.

Litter #	Dog #	Sex	Status	Age of onset (weeks)	Age at exam (weeks)	Weight (kg)	MRI evaluation	Relative cerebellar weight (%)	Overall severity of cerebellar degenerative changes
1	1	m	ataxia	12	16	14	affected	-	moderate
2	2	m	ataxia	8	14	13	affected	-	moderate
	3	m	ataxia	12	14	12	affected	-	moderate
	4*	f	ataxia	-	-	-	-	-	severe
3	5	m	ataxia	4	11	13	affected	-	moderate
4	6	f	ataxia	8	12	9	normal	-	moderate
5	7	m	ataxia	5	14	10	affected	8.3	moderate
	8	m	ataxia	11	14	11	affected	9.3	moderate
	9	m	healthy	-	14	17	normal	-	-
6	10	f	ataxia	10	14	11	affected	9.7	severe
	11	f	ataxia	10	14	11	affected	8.1	severe
	12	f	ataxia	10	14	10	affected	9.7	severe

*: For this one affected puppy, only the brain was received for pathological examination.

MRI = magnetic resonance imaging.

### MRI and histopathological examinations reveal cerebellar neurodegeneration

**Figure 1:**
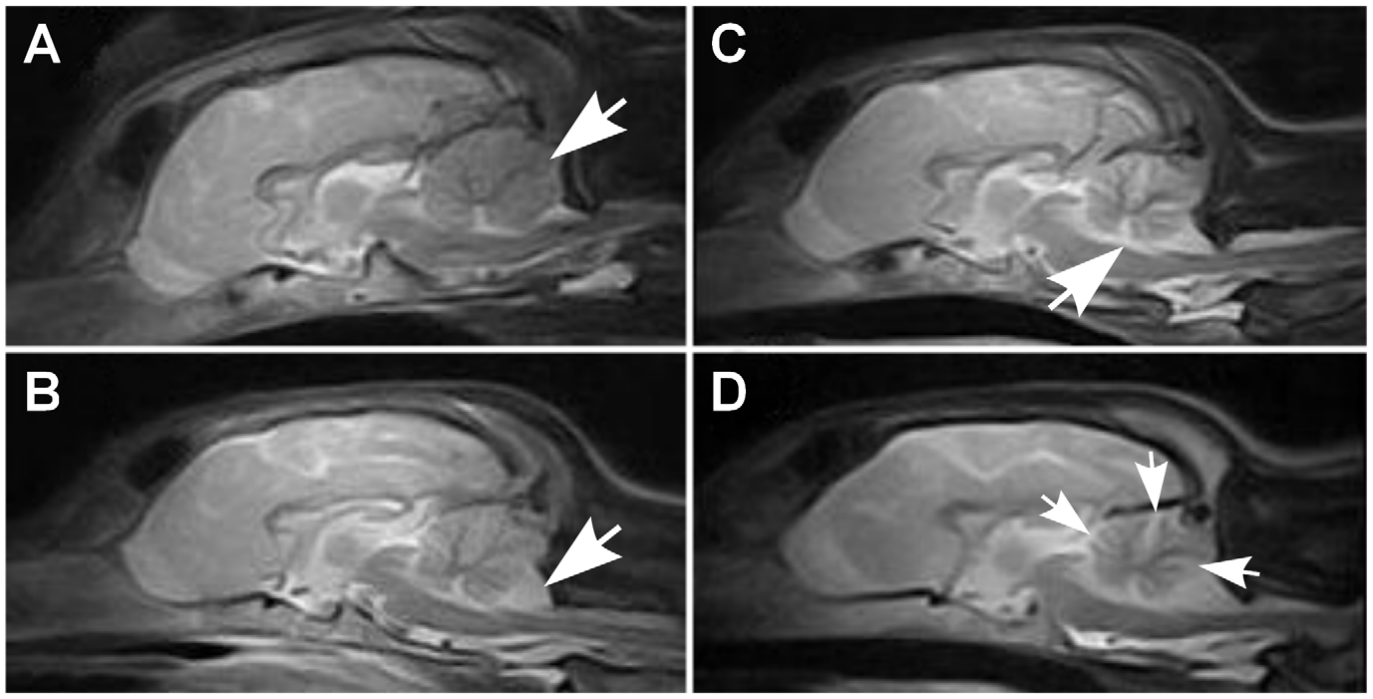
Brain MRI scans. Sagittal midline T2W brain MRI images of a normal and affected Finnish Hounds. (A) A control dog with normal cerebellum (arrow). (B) An affected dog that has smaller cerebellar size and increased cerebrospinal fluid space (white) between the cerebellum and the occipital bone ventrally (arrow). (C) An affected dog that shows reduced cerebellar size and increase in the fluid filled space in the area of the fourth ventricle (arrow). (D) Another affected dog that has reduced cerebellar size and increased fluid filled spaces between the cerebellar folia (arrows).

Cerebellar pathology was supported by magnetic resonance imaging (MRI) and post-mortem examinations. Nine out of ten affected puppies showed reduced cerebellar size on T1- and T2-weighted (T1W and T2W) midsagittal brain MRI scans (Figure 1). No changes were detected in the cerebrum or brainstem. General pathological examination did not reveal any significant gross changes outside the CNS. The weight of the cerebellum relative to the total brain mass was measured in five dogs and ranged from 8.1 to 9.7%. This indicated a loss of cerebellar mass as the normal proportion of the cerebellum is ≥10% [32]. A few disease nonspecific histological findings were made; a mild interstitial pneumonia was seen in four dogs and mild follicular hyperplasia in the spleen of three dogs.

**Figure 2:**
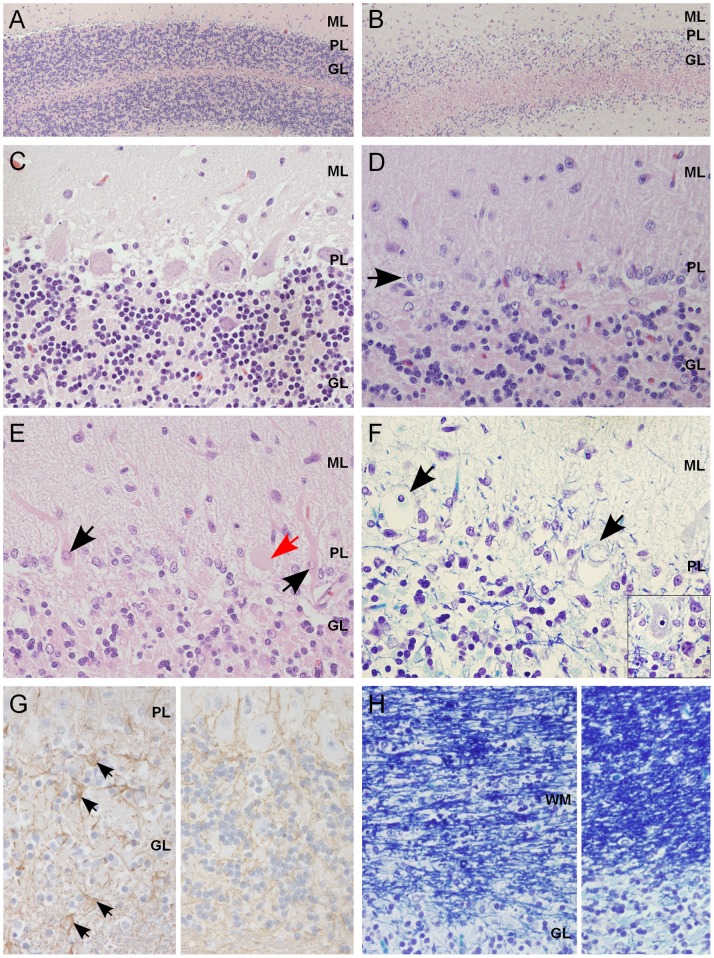
Histological findings within the cerebellar cortex of affected dogs. (A) Normal cerebellar cortex in the ventrolateral parts of the cerebellum, with densely cellular granular cell layer (HE 100×). (B) Affected cerebellar cortex in the vermal region with marked loss of granular cells (HE 100×). (C) A higher magnification of unaffected cortex with viable Purkinje cells (PCs) and a normal granular cell density (HE 400×). (D) A higher magnification of affected cortex, with severe loss of PCs and linear reactive gliosis between the molecular and the granular layers (arrow) (HE 400×). (E) Degenerating, shrunken, eosinophilic PCs with margination of the nuclear chromatin (black arrow) or central chromatolysis of the cytoplasma (red arrow) (HE 400×). (F) PCs that show total loss of cytoplasmic basophilia (Nissl substance) and pyknotic or karyorhektic nuclei (arrows). Inset: viable PC with intact Nissl substance seen as basophilic cytoplasmic granulation (LFB-CEV, 400×). (G) Left: mild astrogliosis (arrows) within the granular cell layer and the white matter of the cerebellar folia. Right: unaffected part of same dog (IHC GFAP, 400×). (H) Left: secondary degeneration and myelinophagia in the affected cerebellar white matter. Right: unaffected parts of same dog (LFB-CEV, 400×). ML = molecular layer, PL = Purkinje cell layer, GL = granular cell layer, WM = white matter.

Histological changes of the nervous system were restricted to the cerebellum in all examined puppies. The cerebellar cortex showed marked premature degeneration and loss of PCs with consequent neuronal depletion in the granular cell layer (Figure 2B and 2D). The cerebellar vermis and the paramedian lobule were consistently the most severely affected areas. The cranial regions of the cerebellar cortex were more affected than the caudal regions. The ventrolateral parts, including paraflocculi and flocculus, were spared and partially normal (Figure 2A and 2C). In the cortical areas, where severe PC loss was present, glial cells (Bergmans glia) were proliferating between the molecular and the granular cell layers (Figure 2D). The remaining PCs were shrunken and eosinophilic with marginated nuclear chromatin or showed total loss of cytoplasmic basophilic Nissl substance (chromatolysis) (Figure 2E and 2F). The granular cell layer was markedly depleted of neurons and showed mild astrocytosis in areas of profound PCs loss (Figure 2G). Occasional degenerated and vacuolated axons were detected in the granular layer. Mild to moderate ongoing degeneration and myelinophagia was seen within the cerebellar white matter of the severely affected areas (Figure 2H). Neither transsynaptic degeneration in the cerebellar nuclei nor retrograde degeneration of the olivary nucleus was found. Immunohistochemical (IHC) staining for canine distemper virus and parvovirus showed no positivity. The overall severity of the histopathological findings, including active PC degeneration, total granule cell and PC loss, consecutive white matter lesions and the extent of the lesions, are summarized in Table 1.

### Genetic studies map a novel recessive ataxia locus

**Figure 3:**
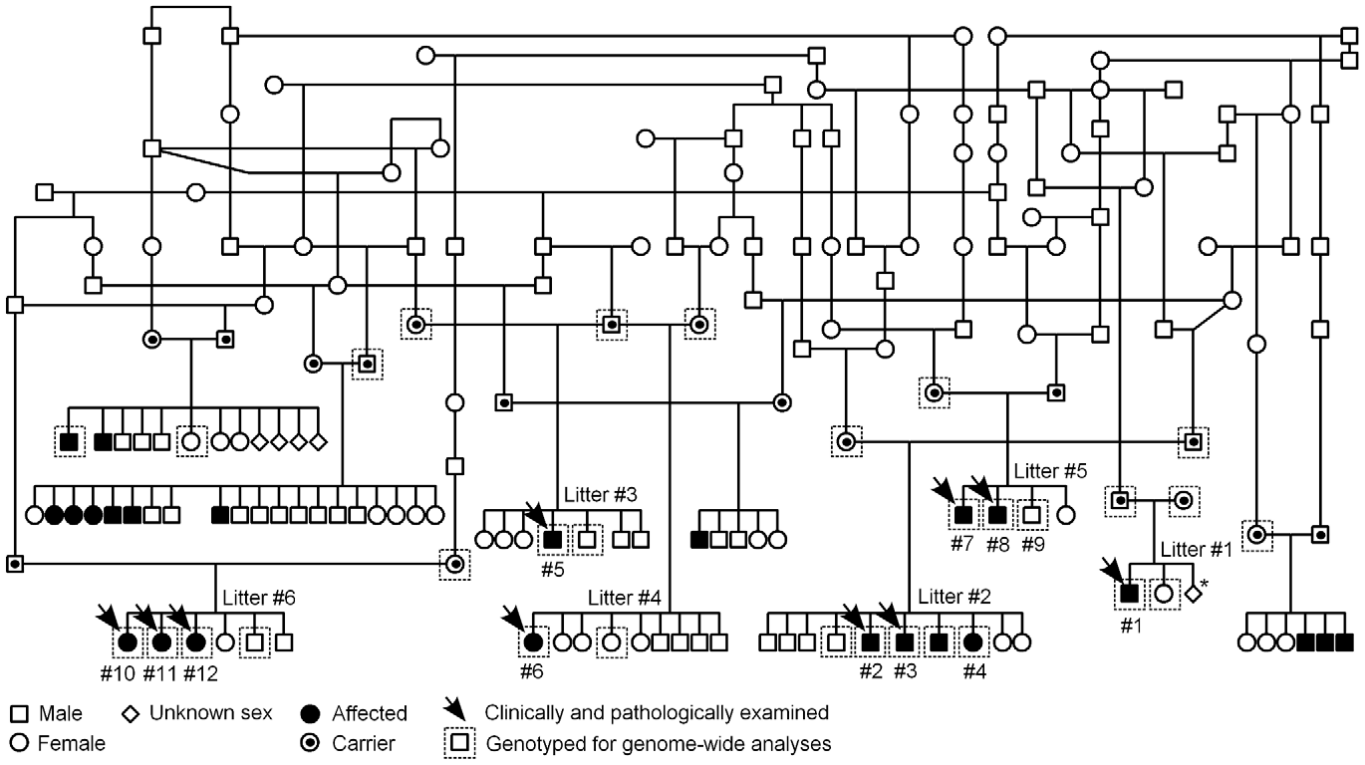
Finnish Hound ataxia pedigree. Pedigree shows those affected litters that were used in the study. Disease segregation is consistent with autosomal recessive mode of inheritance as all affected dogs are born from healthy parents and both sexes are affected. The proportion of affected puppies is 29%, which is close to expected 25%. Denoted are the clinically and pathologically examined cases and the dogs that were genotyped for genome-wide analyses. The numbering of litters and puppies refers to the numbering used in Table 1. *In this one litter, the total number of offspring was unknown.

A pedigree was established around the known affected FH puppies to determine the most likely mode of inheritance (Figure 3). According to the pedigree data, all affected dogs were born from healthy parents, both sexes were affected and the proportion of affected puppies across litters was 29%, near the expected 25%. These observations were consistent with an autosomal recessive mode of inheritance.

We first used a candidate gene approach to try to identify the causative gene. Altogether 24 known human and murine ataxia genes were selected (Table S1), and the segregation of microsatellite markers, within or adjacent to the candidate genes, was studied in three nuclear families. Each family included both parent dogs and at least one affected and one healthy offspring. No co-segregation was observed between any of the markers and the disease.

**Figure 4:**
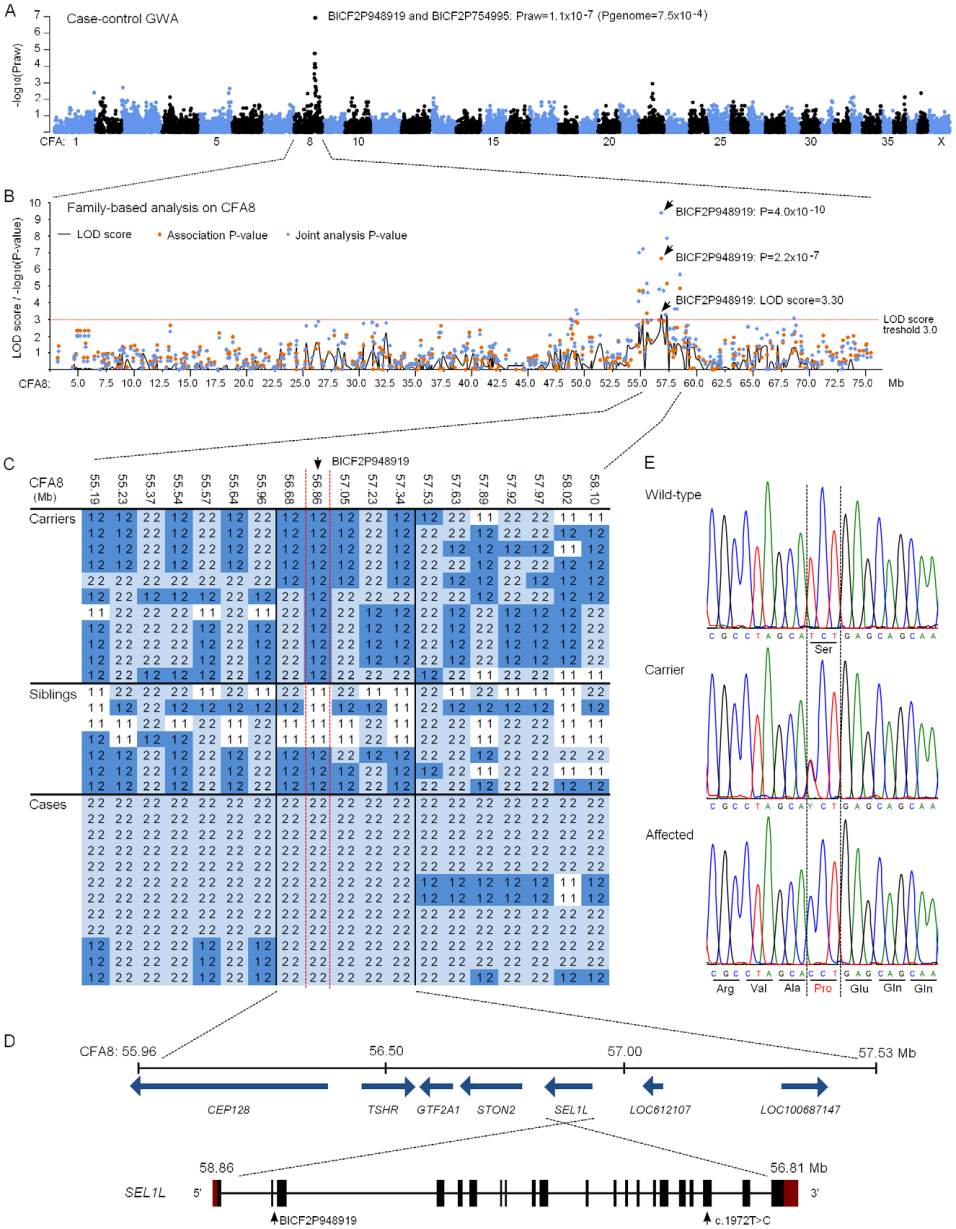
Genetic studies. (A) A Manhattan plot of case-control genome-wide association test performed using 13 cases and 7 unaffected sibling-controls. (B) Results of the family-based testing on the disease associated chromosome 8. Plotted are single-point linkage analysis LOD scores, association test p-values and joint analysis p-values. (C) Genotypes at the disease associated locus on CFA8. All cases share a 1.5 Mb homozygous block, and within this block BICF2P948919 shows complete segregation with the disease. (D) A schematic representation of the seven genes found on the 1.5 Mb block and of the *SEL1L* gene structure. *SEL1L* exons are marked with black boxes and red denotes the untranslated regions (UTRs). BICF2P948919 is located on second *SEL1L* intron and the c.1972T>C mutation on exon 19. (E) Chromatograms of the c.1972T>C mutation in an affected, a carrier and a wild-type dog.

We subsequently proceeded to map the FH ataxia locus by using a genome-wide approach. A cohort of 31 dogs, comprising 13 cases, 11 obligate carrier parents and seven non-affected siblings, were genotyped using Illumina's 22K canine SNP chip. A standard case-control association test was carried out on the 13 cases and seven full-sibling controls by using PLINK software [36]. This revealed a genome-wide significant association on CFA8 with two SNPs, BICF2P948919 and BICF2P754995, that had the best nominal and corrected p-values of p_raw_ = 1.1×10^−7^ and p_genome_ = 7.5×10^−4^ (Figure 4A). The association results were confirmed by utilizing a joint family-based linkage and association analysis program PSEUDOMARKER [37]. The joint analysis, which was carried out on the associated CFA8 using the entire genotyped sample cohort, identified the same locus and a single most significant SNP, BICF2P948919 (LOD score = 3.3, association p = 2.2×10^−7^ and joint analysis p = 4.0×10^−10^) (Figure 4B).

Assessment of genotypes at the associated CFA8 locus revealed a shared 1.5 Mb homozygous haplotype block in affected dogs, spanning from 56.0 to 57.5 Mb (Figure 4C). All genotyped parent dogs were carriers of this disease haplotype. Within the 1.5 Mb block, only the most significant SNP, BICF2P948919, showed complete segregation with the disease, indicating that the causative variant probably lies in its vicinity. The 1.5 Mb haplotype contained seven genes. Two of these, *LOC100687147* (serine/threonine-protein kinase Nek6-like) and *LOC612107* (uncharacterized hypothetical gene) were likely pseudogenes. The other five were known protein-coding genes, centrosomal protein 128kDa (*CEP128*), thyroid stimulating hormone receptor (*TSHR*), general transcription factor IIA (*GTF2A1*), stoning 2 (*STON2*) and sel-1 suppressor of lin-12-like (*SEL1L*) (Figure 4D). We ranked *SEL1L* as the best candidate gene for mutation screening due to its neuronal expression, and function in a protein degradation pathway in the endoplasmic reticulum (ER) [38]–[41]. Impaired protein degradation is a common feature in several neurodegenerative diseases [42], [43]. In addition, the fully segregating SNP, BICF2P948919, was located on the second intron of the *SEL1L* gene. Besides *SEL1L*, the *STON2* gene was considered a plausible causative gene because of its neuronal function in synaptic vesicle recycling at presynaptic nerve terminals [44]–[47].

### Exonic sequencing identifies a missense mutation in the *SEL1L* gene

The coding regions of the five known protein coding genes were sequenced in two affected puppies and in two obligate carrier parents in order to identify possible disease-causing mutations. No sequence variants that segregated with the phenotype were found in *TSHR* or *GTF2A1*. Sequencing of *STON2* and *CEP128* revealed a few segregating variants but none of them were in the coding regions (Table S2). In *SEL1L*, we identified altogether four coding and 19 non-coding variants, all of which segregated with the disease (Table S2). Three of the exonic variants were synonymous but one was a non-synonymous cytosine to thymine change on exon 19 (c.1972T>C) that results in a serine to proline alteration at position 658 of the encoded protein (p.Ser658Pro) (Figure 4E). The c.1972T>C variant was genotyped in the full sample cohort and showed complete segregation and a 100% penetrance with the disease. All 13 affected puppies were homozygous for the T>C change, all 13 parents heterozygous and 20 littermates either heterozygous (12 out of 20) or wild-type (8 out of 20). Segregation of the variant was further validated by genotyping altogether 241 randomly selected unaffected FHs. None of these population controls were homozygous for the C allele but a 10% carrier frequency (24/241) was indicated. Segregation analysis gave a highly significant association between the C allele and disease (p = 1.8×10^−42^). Moreover, the c.1972T>C allele was completely absent in 349 dogs from 51 other breeds (Table S3), including a Russian hound breed, which is related to FHs. We later received a sample from a newly affected FH puppy from Finland that was found homozygous for the CC genotype. Another suspected FH ataxia puppy from Sweden was tested free of the mutation. However, further inquiries of this puppy's phenotype revealed that it had not presented with clinical signs typical for FH ataxia but had in reality suffered from an episodic disorder. The puppy could not be examined further as it had been euthanized at 6 weeks of age.

**Figure 5:**
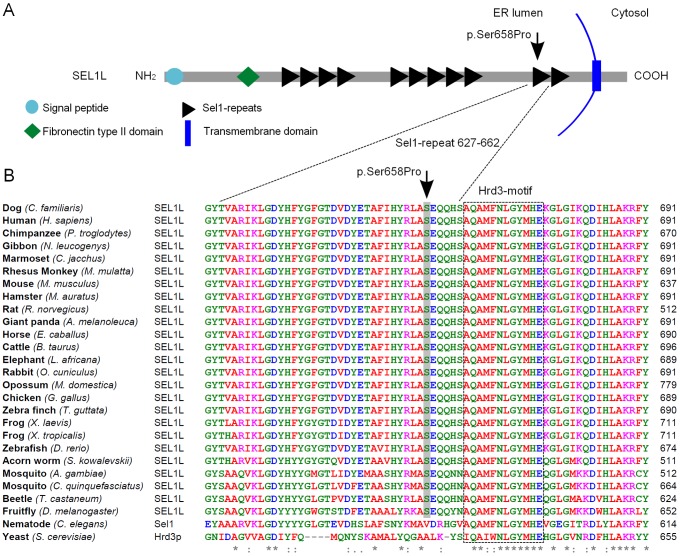
SEL1L protein structure and partial sequence alignment. (A) A Schematic representation of the SEL1L protein, which shows the multi-modular protein structure. The p.Ser658Pro mutation hits one of the C-terminal Sel1-repeats. (B) SEL1L amino acid sequence alignment. Position of the mutation is conserved in all aligned species except for *C. elegans* and yeast. The Hrd3-motif, which is positioned five residues downstream from the Ser658, shows conservation across all aligned species.

The probability of the SEL1L p.Ser658Pro change having a pathogenic effect was evaluated by utilizing bioinformatics tools. SEL1L is a transmembrane glycoprotein that resides in the endoplasmic reticulum (ER) [48], [49]. The carboxy-terminus of the protein harbors the transmembrane domain and amino-terminal protein body is exposed to the ER lumen. The lumenal protein body is composed of a single fibronectin type II domain and three clusters of tetratricopeptide repeat (TPR)-like motifs, the Sel1-repeats (Figure 5A) [39], [40], [49], [50]. The p.Ser658Pro amino acid change is positioned in one out of the several Sel1-repeat motifs of the protein (Figure 5A). We found Ser658 to be fully conserved in all aligned vertebrates, insects and in the hemichordate acorn worm. Only *C. elegans*'s orthologue Sel1 and yeast's orthologue Hrd3p differed at the position (Figure 5B). Moreover, three sequence homology-based prediction tools, PANTHER, PolyPhen and SIFT, all predicted the Ser658Pro change to have a probably damaging effect on protein function (PANTHER SubPSEC score = −5.4, PolyPhen-2 (HumVar-trained) score = 0.98 and the SIFT score = 0.02).

**Figure 6:**
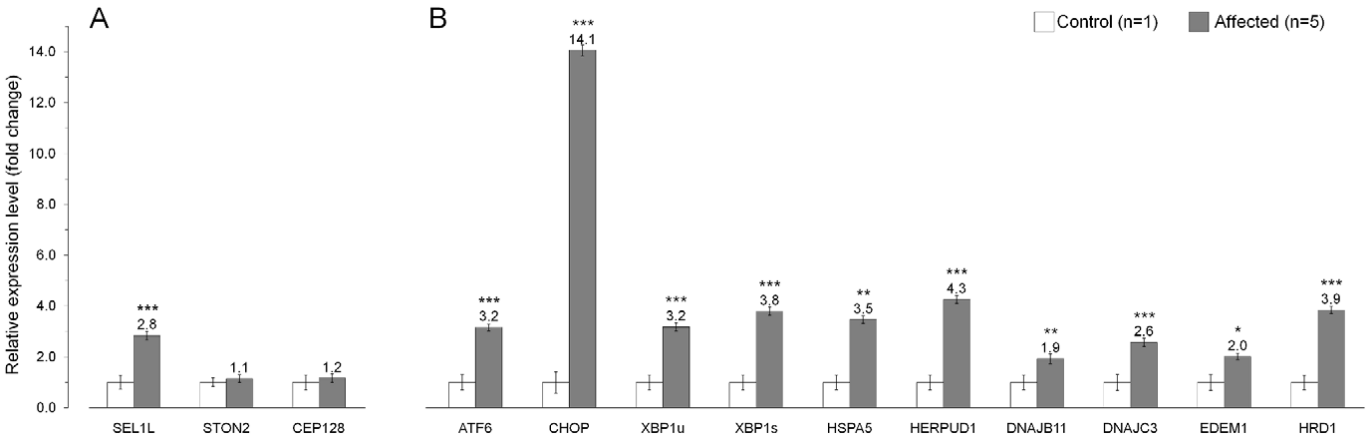
Gene expression analysis in the affected cerebellum. (A) The mRNA levels of *SEL1L*, *STON2* and *CEP128* in the cerebellar cortex of five affected dogs compared to a control puppy. These three genes had sequence variants that segregated with the ataxia phenotype. (B) Endoplasmic reticulum (ER) stress markers are upregulated in the cerebellar cortex of affected animals. The relative mRNA expression levels are represented as a fold change. Error bars denote the standard error of normalized Ct-values. *p≤0.05 **p≤0.001 ***p≤0.000 (two-tailed t-test p-values).

According to various expression databases, mammalian *SEL1L* is ubiquitously expressed. However, we wanted to specifically confirm *SEL1L* cerebellar expression and also to see, if the non-synonymous c.1972T>C variant has an effect on *SEL1L* mRNA stability. Amplification and sequencing of the entire *SEL1L* transcript confirmed cerebellar expression, the presence of the mutation at mRNA level and the predicted exon/intron boundaries, and finally, excluded the possible splicing effects of the several identified intronic variants (Table S2). Real-time quantification of the *SEL1L* transcript in cerebellar cortical tissue samples of five affected dogs and a control puppy revealed a 2.8-fold increase in the affected dogs (Figure 6A). We also studied the cerebellar expression of the two other genes with segregating non-coding variants, *STON2* and *CEP12*, but did not find differences in their transcript levels between the control and affected dogs (Figure 6A).

### Upregulation of ER stress genes in affected cerebellar cortex

SEL1L is a component of an ER-associated protein complex that functions in protein degradation [38]–[41]. Impaired protein degradation is known to affect ER homeostasis and result in ER stress [51], [52] We therefore hypothesized that the c.1972T>C change in *SEL1L* could lead to dysfunction of the protein complex and cause ER stress. Disruption of ER homeostasis activates the unfolded protein response (UPR), which upregulates genes that are required for cell survival during ER stress [53]–[57]. We measured the transcript levels of nine known ER stress responsive genes, *ATF6*, *CHOP*, *XBP1*, *HSPA5*, *HERPUD1*, *DNAJB11* (ERdj3), *DNAJC* (p58^IPK^), *EDEM1* and *HRD1* in cerebellar cortical tissue of five affected FHs and a control dog. For *XBP1*, we measured both the unspliced (XBP1u) and the spliced (XBP1s) transcripts [58]–[60]. All tested UPR genes showed increased expression levels in the affected dogs (Figure 6B). The highest, 14-fold increase was observed for *CHOP*, whereas 1.9 to 4.2-fold increases were observed for the other eight UPR genes. These results are indicative of ER stress in the affected cerebellar cortex and support the pathogenicity of the identified *SEL1L* mutation.

## Discussion

We describe here the clinical and histopathological phenotype of a progressive early-onset cerebellar ataxia in FHs and identify a missense mutation in the *SEL1L* gene that segregates with the recessive disease. The clinical course in FH ataxia is compatible to the classical canine cerebellar abiotrophy, which has an early-onset, rapid progression and poor prognosis [16], [17], [19]–[22], [25], [27], [28]. Affected FHs present with a progressive ataxia that causes significant ambulatory difficulties. The symptoms worsen rapidly and the affected puppies are euthanized soon after diagnosis. Cerebellar degeneration is visible in MRI at the age of 3 months. Histopathological features are consistent with a premature degeneration of cerebellar cortical PCs, where the PCs are the primary target of a degenerative pathological process, followed by secondary changes in other cortical cell layers. We suggest that the now identified *SEL1L* mutation (c.1972T>C, p.Ser658Pro) is the most likely underlying cause in FH ataxia. Several lines of evidence support *SEL1L* as the causative gene.

First, *SEL1L* is the best positional and functional candidate in the 1.5 Mb disease associated haplotype block. The haplotype contained five known protein coding genes, which were screened for mutations. The only coding variant that causes a protein level change was found in the *SEL1L* gene. *SEL1L* shows ubiquitous expression in adult tissues and is widely expressed during embryonic development, with intense expression in developing neural tissue and pancreas [49], [61], [62]. We confirmed *SEL1L* expression in the cerebellar cortex, the affected organ in FH ataxia.

Second, the SEL1L protein belongs to a disease-relevant pathway. SEL1L functions in a large protein complex in the ER, in a cellular process referred to as ER-associated degradation (ERAD) [38]–[41]. The ERAD machinery targets terminally misfolded and unassembled polypeptides which are recognized, dislocated to the cytoplasm and marked for proteasome-dependent degradation [55], [63], [64]. SEL1L is a component in an ERAD complex that is organized around an E3 ubiquitine ligase, HRD1 [38], [40], [41], [65]–[67]. Although the precise function of SEL1L is not known, it is proposed to have an adaptor role and to be involved in recruiting ERAD substrates to the HRD1 ligase complex, either directly or via other proteins [39], [40], [65]–[67]. ER stress and impaired protein degradation have been implicated in several neurodegenerative diseases [52], [68], [69]. As an indication of acute ER stress in the cerebellar cortex of affected FHs, we show increased expression of several transcription factors and chaperone proteins that belong to the ER stress response pathway, UPR. Increased transcription levels were also found for SEL1L and HRD1, which are both known to be upregulated in ER stress [70]. However, the highest increase was detected in the expression of a proapoptotic transcription factor CHOP [71]–[73]. CHOP is normally expressed at very low levels but is highly induced in ER stress [74], [75]. Overall, our expression data supports the pathogenic role of the *SEL1L* mutation in FH ataxia. There is one previous report that connects *SEL1L* to a neurodegenerative phenotype. A tentative association was found between an intronic *SEL1L* SNP and Alzheimer's disease [76]. However, given the early-onset and rapid progression of the disease in FHs, our study indicates that SEL1L has crucial role in the developing brain and suggests that *SEL1L* is an unlikely candidate gene for late-onset neurodegenerative disorders. This is further supported by a prenatal lethality of Sel1l-deficient mice [77].

Third, the p.Ser658Pro amino acid change hits a highly conserved residue in an evolutionary conserved repeat motif in the SEL1L protein. The affected domain is the next to last carboxy-terminal Sel1-repeat. Unlike the amino-terminal fibronectin type II domain, which is absent from invertebrate SEL1L orthologues, the Sel1-repeats are highly conserved across species [49]. The α/α-helical Sel1-repeats are found in several proteins that function as adaptors in protein complex formation [78]. Deletion of the two most carboxy-terminal Sel1-repeat clusters in mammalian SEL1L have been reported to abolish interactions with HRD1 and other proteins [40], and recent data suggests that the stabilization of the mammalian SEL1L depends on HRD1 [79]. Sequence homology-based prediction programs all indicated a likely damaging effect of the p.Ser658Pro change. Moreover, there is a considerable difference in structure between the two amino acids, serine and proline. The latter possesses a conformationally restricted cyclic molecular structure, which could interfere with proper protein folding. Given the role of the Sel1-repeats in protein interactions, it is possible that the p.Ser658Pro change disrupts SEL1L protein-protein interactions, for instance with HRD1, compromising the function of the ERAD complex.

Forth, a mouse model connects Sel1l deficiency to impaired ER-mediated protein quality control. A recent paper reported embryonic lethality and signs of systemic ER stress in Sel1l-deficient mice [77]. Heterozygous mice were normal and fertile but those that were homozygous for a gene trap mutation between *Sel1l* exons 14 and 15, died at mid-gestation, and suffered from growth retardation and morphological brain abnormalities [77]. Cell lines derived from the mutant mice revealed changes in ER morphology, hypersensitivity to ER stress inducers, defects in degradation of unfolded proteins and an impaired protein secretory pathway [77]. In accordance with our results, several UPR genes were upregulated in the mutant embryos [77]. The embryonic lethality of the Sel1l-deficient mice suggests that the canine mutation is milder and does not abolish all SEL1L function. The affected FH puppies did not show any gross morphological abnormalities and seemed to develop normally over the first few weeks of life. Furthermore, some SEL1L functions might be complemented by protein isoforms [49], [62], [80]–[82]. Amino-terminal SEL1L isoforms that contain a variable number of Sel1-repeats but lack the carboxy-terminal region, have been indicated to function in parallel or complementary to the full length SEL1L in ER stress [81], [82].

Although SEL1L is ubiquitously expressed, another explanation for the cerebellum-restricted pathology may come from the vulnerability of the cerebellar PCs to ER stress. Various disease phenotypes indicate that cerebellar PCs are susceptible to undergo premature degeneration [13], [14], [33], [83], [84] and accumulating evidence indicate ER stress specifically as causative in PC pathology [85]–[92]. These observations could account for the primary PC degeneration in affected FHs. In addition to our results on *SEL1L* cerebellar expression, a previous study found HRD1 in murine PCs [93]. It is plausible that a compromised function of the HRD1-ligase pathway would not be tolerated by ER stress sensitive PC and would lead to excessive or prolonged ER stress, triggering apoptosis and neurodegeneration. Whether any pathological changes would be seen in other tissues if the affected dogs were kept alive longer, is not known.

In conclusion, we identify a novel progressive ataxia gene and link a defective ERAD pathway to an early-onset cerebellar neurodegeneration. Our study implicates a critical function for the HRD1-SEL1L-mediated ERAD pathway in the postnatal PC survival and provides a novel model to investigate the role of the ER stress in neurodegeneration. *SEL1L* represents a novel candidate gene for human ataxias and we have initiated mutation screenings in childhood phenotypes. Meanwhile, a genetic test has been offered for FH breeders to identify carriers and to eradicate the disease from the breed.

## Materials and Methods

### Ethics statement

All the dogs used in this study were privately-owned pets, and the genetic and clinical examinations were approved by the Animal Ethics Committee at the State Provincial Office of Southern Finland (permits: ESLH-2006-08207/Ym-23 and ESHL-2009-07827).

### Animals and blood samples

EDTA-blood samples were collected from 13 affected FH puppies that belonged to seven different litters, and from 33 unaffected first-degree relatives. The unaffected relative sample cohort comprised 13 parent dogs and 20 unaffected full-siblings. A pedigree was constructed around the affected dogs by using GenoPro genealogy software (http://www.genopro.com/). FH population controls (n = 241) and an additional control sample cohort of 349 dogs from 51 other dog breeds were selected among samples stored at the Canine DNA Bank located at Biomedicum Helsinki, Finland. The FH population cohort included random samples from unaffected FHs that had been collected for instance at dog shows. Full-siblings were excluded from the population cohort to obtain a reliable estimate a carrier frequency. The control cohort from other breeds comprised one dog from two breeds, two dogs from 38 breeds and 10 to 32 dogs from 11 breeds (Table S3). Puregene DNA Purification Kit (Gentra Systems) was used to extract genomic DNA from affected dogs and their relatives. A semi-automated Chemagen extraction robot (Chemagen Biopolymer-Technologie AG) was used for the control samples. Concentration of DNA samples was determined using a ND-1000 UV/Vis Spectrophotometer (NanoDrop Technologies).

### Clinical examinations

General clinical, orthopedical and neurological examinations were performed on ten ataxic FH puppies that were referred to a veterinary neurology clinic during December 2006 and September 2008. The examined puppies came from six different litters. One healthy sibling was examined as a control dog. Neurological examinations were filmed and are available for retrospective evaluation. CBC and serum biochemistry profiles (sodium, potassium, calcium, phosphorus, magnesium, glucose, total protein, albumin, globulin, blood urea nitrogen, creatinine, total bilirubin, alanine aminotransferase, aspartate aminotransferase, alkaline phosphatase, and creatine kinase) were examined on admission. Brain MRI scanning and CSF sample collection were performed under general anesthesia. Intramuscular anaesthesia premedication was carried out with the combination of Medetomidine (Domitor, Orion Pharma) 0.02 mg/kg and Butorphanol (Torbugesic, Scan-Vet) 0.1 mg/kg. Anaesthesia was induced through intravenous bolus of Propofol (Propofolum, Abbott Laboratories) 6–8 mg/kg and maintained through inhalation of Isoflurane (IsoFlo vet, Orion Pharma) and oxygen.

A 0.2 Tesla MRI scanner (VetMR, Esaote) was used to record T1W and T2W images in transversal and sagittal planes on animals placed in sternal recumbency. TW1 images were recorded using 750.0 ms repetition time (TR), 26.0 ms echo time (TE) and a field of view (FoV) of 150×150, 160×160 and 170×170 mm and T2W images with 3000.0 ms TR, 90.0 ms TE and 160×160 or 170×170 mm FoV. In all images, slice thickness was 4.0, 4.5 or 5.0 mm and interslice gap 0.4–0.5 mm. T1W images were repeated immediately after intravenous injection of contrast agent, gadolinium-diethylenetriaminepenta-acetate (-DTPA) dimeglumine 0.2 mL/kg (0.1 mmol/kg). Two blinded examiners (SC and JJ) rated the dogs subjectively as affected or healthy based on cerebellar size, amount of CSF between the cerebellar folia, size of the fourth ventricle and distance between caudoventral edge of the cerebellum and foramen magnum. CSF samples were collected from the cerebellomedullary cistern after the MRI examination. Total cell count and protein concentration were evaluated from the CSF samples and considered normal if there were less than five nucleated cells per microliter and if Pandy reaction was negative. Affected dogs with compatible clinical signs and MRI findings were euthanized with owner's agreement.

### Pathological and histological examinations

A complete autopsy was performed on the ten clinically examined dogs. For one additional puppy, only the brain was received for examination. The weight of the cerebellum relative to the total weight of the brain was determined in five dogs. Samples from the CNS, liver, lungs, spleen, kidney and heart were collected and fixed in neutral buffered 10% formalin. Sections from the fixed tissues were embedded in paraffin and processed for light microscopic examination, using the haematoxylin-eosin (HE) stain. The CNS was sectioned at nucleus caudatus with cerebral cortex, hippocampus with temporal cortex, mesencephalon at the height of the cranial colliculi, cerebellum transversally and longitudinally with pons and medulla oblongata. Sections from the CNS were stained with luxol fast blue/cresyl echt violet (LFB-CEV) to evaluate chromatolysis and myelin loss. An IHC stain for glial fibrillary acidic protein (GFAP, MCA 1909, Serotec) was used to assess astrogliosis. IHC staining of spleen, lung and kidney sections were performed for canine distemper virus (CDV, MCA 1893, Serotec), and sections of spleen were stained for canine parvovirus (CPV, MCA2064, Serotec).

### Candidate gene analysis

Altogether 24 known human and murine ataxia genes were selected for a microsatellite marker-based candidate gene analysis (Table S1). Segregation of microsatellite markers was examined in three nuclear families, comprising six parents, five affected dogs and three healthy siblings. Allele sizes were determined by fragment analysis. Human and murine mRNA sequences for the candidate genes were obtained from the GeneBank database (http://www.ncbi.nlm.nih.gov/) and the corresponding canine sequences were identified from the CanFam2.0 annotation using the BLAT search tool [94]. Microsatellite primers (Table S4) were designed using Primer 3 (http://frodo.wi.mit.edu/primer3/). Forward primers were either directly labeled with a fluorescence dye (HEX or FAM) or alternatively, an M13-tail sequence was added to the 5′-end and used together with a third, FAM-labeled M13-primer [95]. PCR amplifications were performed using a PTC-225 Peltier Thermal Tetrad Cycler (Bio-Rad) and a standard PCR protocol. Reactions that included directly labeled forward-primers were performed in a reaction volume of 10 µl with 20 ng of genomic DNA, 1 X PCR buffer, 2.5 mM MgCl_2_, 0.2 mM dNTPs, 0.5 µM of forward- and reverse primers and 0.375 units of AmpliTaq Gold Polymerase (Applied Biosystems). Amplifications that were performed using the M13-primers were carried out in a 12 µl volume containing 15 ng of genomic DNA, 1 X PCR buffer, 2.1 mM MgCl_2_, 0.33 mM dNTPs, 0.05 µM M13-tailed forward primer, 0.25 µM reverse primer, 0.2 µM M13 primer and 1.2 units of Biotools DNA polymerase. Intensity of PCR products was evaluated from 1% agarose gel stained with 0.5 µg/ml ethidium bromides (Amresco). Fragment analysis runs were performed on a 3730xl DNA Analyzer (Applied Biosystems). The Peak Scanner software (Applied Biosystems) was used to determine allele sizes.

### Genotype data analysis

Thirteen affected dogs from seven nuclear families and 18 related control dogs were genotyped using Illumina's CanineSNP20 BeadChip of 22,362 validated SNPs. Healthy control dogs included 11 obligate disease carrier parents and seven non-affected siblings (Figure 3). Genotype data was filtered using a SNP call rate of >95%, an array call rate of >95% and minor allele frequency of >5%. Based on these criteria, 289 SNPs were removed for low genotyping efficiency and 6739 SNPs for low minor allele frequency. Mendel errors were detected in 35 SNPs, which were removed from analyses. No samples were removed for low genotyping and no SNPs for significant deviations from the Hardy-Weinberg equilibrium (p≤0.0001). After the filtering steps, 15,299 SNPs remained for analyses. A basic case-control association test was performed by using the software package PLINK [36]. Obligate carrier parents were excluded from the case-control association test and the remaining seven healthy siblings were used as controls. Genome-wide significance was ascertained through phenotype permutation testing (n = 100,000). Pseudomarker program was used to perform family-based testing on CFA8, which showed genome-wide significant association in the PLINK analysis [37]. The family-based analyses were performed under a recessive inheritance model, and included parametric single-point linkage test, association analysis (LD|Linkage) and joint analysis (LD+Linkage).

### Mutation screening

Mutation screening of *CEP128*, *TSHR*, *GTF2A1*, *STON2* and *SEL1L* exons, exon-intron junctions and 5′ and 3′ UTRs was performed using samples from two affected dogs and two obligate disease carriers. Primers (Table S5) were designed by using Primer 3 (http://frodo.wi.mit.edu/primer3/). PCR reactions were carried out in a total reaction volume of 20 µl with 20 ng of genomic DNA, 1 X PCR buffer, 2 mM MgCl_2_, 0.2 mM dNTPs, 0.5 µM of forward- and reverse primers and 0.5 units of Biotools DNA Polymerase. PCR amplification was performed using a PTC-225 Peltier Thermal Tetrad Cycler (Bio-Rad) and a standard PCR protocol. The reaction products were run on a 1% agarose gel stained with 0.5 µg/ml ethidium bromide (Amresco). PCR products were purified with ExoSAP-IT (GE Healthcare) and sequenced using an Applied Biosystems' 3730xl DNA Analyzer. Sequence Scanner v1.0 and Variant Reporter v1.0 (Applied Biosystems) were used to assess sequence quality and identify polymorphisms. Control sample cohorts were screened by using Applied Biosystems' TaqMan chemistry and 7500 Fast Real-Time PCR instrumentation. The genotyping reactions were carried out in a 10 µl reaction volume with 1 X TaqMan genotyping assay (Applied Biosystems), 1 X Taqman Genotyping Master Mix (Applied Biosystems) and 10 ng of genomic DNA. Primer sequences for the Taqman assay were 5′- CGTAGACTACGAGACTGCATTTATTCA -3′ for the forward, and 5′ - GATTAAACATAGCTTGTGCACTGTGT - 3′ for the reverse primer. Probe sequences were 5′ - TGCTGCTCAGATGCTA - 3′ and 5′ - TGCTGCTCAGGTGCTA - 3′, labeled with VIC and FAM, respectively.

### Bioinformatic analysis

Pfam protein families database (http://pfam.sanger.ac.uk/) [96] and the SMART tool (http://smart.embl-heidelberg.de/) [97], [98] were used to confirm the protein domain structure of canine SEL1L. The ClustalW2 algorithm was used to compose a multiple sequence alignment to examine cross-species conservation (http://www.ebi.ac.uk/Tools/clustalw2/). Three sequence homology-based software tools, PANTHER (http://www.pantherdb.org/tools/) [99], [100], PolyPhen-2 (http://genetics.bwh.harvard.edu/pph2/) [101] and SIFT (http://sift.jcvi.org/) [102]–[104], were used to predict the potential functional impact of the identified non-synonymous variant. The PANTHER tool has a score range from 0 to about −10, with a cutoff for functional significance at ≤−3. The PolyPhen-2 score ranges from 0 to 1, with the threshold for probably damaging at 0.85. The SIFT score ranges from 0 to 1, and substitutions are predicted to affect function if the score is ≤0.05.

### Tissue samples and mRNA experiments

Tissue samples were collected from the cerebellar cortex of five affected puppies immediately after euthanasia. Samples were stabilized in RNAlater reagent (Ambion, Inc) and stored in −80°C. Total mRNA extraction was performed using the RNeasy Mini Kit (Qiagen) with a DNase I digestion step included (RNase-Free DNase Set, Qiagen). Concentration of total RNA was measured using a ND-1000 UV/Vis Spectrophotometer (NanoDrop Technologies). Reverse-transcriptase (RT) -PCR was carried out on equal amounts of RNA in each sample by using the High Capacity RNA-to-cDNA Kit (Applied Biosystems). A cerebellar control sample was obtained from an 11 days old Saluki puppy that was put down due to a peritoneo-pericardial hernia.

*SEL1L* mRNA sequencing primers (Table S5) and all qPCR primers (Table S6) were designed using Primer 3 (http://frodo.wi.mit.edu/primer3/). If possible, forward and reverse primers were positioned in different exons to help control for genomic DNA contamination. *SEL1L* mRNA amplification reactions and sequence analysis were performed as described for mutation screening. Real-time quantitative PCR was performed by using the Applied Biosystems' 7500 Fast Real-Time PCR instrumentation and Roche's FastStart Universal SYBR Green Master. A total reaction volume of 20 µl was used, together with a 0.25 µM concentration of forward- and reverse primers. Two house-keeping genes, GAPDH and YWHAZ, were used as normalization controls, and triplicate samples were used for all reactions. The efficiency of the qPCR reactions was calculated from a five-point dilutions series. No significant differences were detected in the efficiencies between the house-keeping and target reactions, and the comparative ΔΔCt-method could be used to determine relative expression differences [105]. Statistical significance of the expression differences was calculated by using the Student's t-test on normalized mean cycle threshold (Ct) -values. PASW Statistics 18 software (IBM) was used to perform the statistical tests.

## Supporting Information

Table S1 Ataxia candidate genes.
(PDF)

Table S2 Variants identified in candidate gene sequencing.
(PDF)

Table S3 Control dogs from other breeds.
(PDF)

Table S4 Microsatellite marker primers.
(XLSX)

Table S5 Candidate gene sequencing primers.
(XLSX)

Table S6 Primers for quantitative PCR.
(XLSX)

Video S1 Gait. Video shows the normal gait of an unaffected three months old Finnish Hound puppy and the altered gait of two affected similarly aged puppies. Note the difficulty of the affected puppies to control limb movements (ataxia), particularly in the hind limbs. Affected puppies present with swaying of the whole body (truncal ataxia) and abnormal postures, which are caused by the coordination difficulties. The puppies struggle with movement coordination, especially when the direction or speed of the movement changes or when the movement is initiated or stops abruptly. This is caused by inability to control the distance and scale of the movement (dysmetria), which leads to too short or too long steps.
(WMV)

Video S2 Postural reactions. Video shows postural reactions in an unaffected and affected puppy. Postural reactions were examined by testing the puppies' proprioception (overknuckling), hopping reaction and postural thrust reaction, and by performing the wheelbarrowing test (tests appear in this order in the video). Proprioceptive positioning is normal in both the healthy and affected puppy as cerebellum is not involved in this reaction. In the other three tests, the affected puppy shows delayed initiation and dysmetria when it is stepping or jumping.
(WMV)

Video S3 Intention tremor. Video shows four affected puppies that suffer from intention tremor, which is a type of tremor never seen in healthy puppies. The tremor is predominantly seen when movement is initiated (first puppy) or when there is an intention to eat or smell (other three puppies). The amplitude of the intention tremor of the head varies from low (second and third puppy) to high (fourth puppy).
(WMV)
