# Supplementary material for: A SEL1L Mutation Links a Canine Progressive Early-Onset Cerebellar Ataxia to the Endoplasmic Reticulum–Associated Protein Degradation (ERAD) Machinery
Source: PLoS Genet. 2012 Jun 14;8(6):e1002759. doi: 10.1371/journal.pgen.1002759 (PMC3375262; doi:10.1371/journal.pgen.1002759)
Supplement: Table S1 — Ataxia candidate genes. (PDF) [file pgen.1002759.s001.pdf]

Table S1. Ataxia candidate genes.

	Gene	GenBank ID	Locus in human / mouse (Mb)	Locus in dog (Mb)	Human disease / Mouse mutant, Inheritance	Reference
**Human disease**	*APTX*	54840	Chr9: 32.96-32.99	Chr11: 53.14-53.15	Ataxia with oculomotor apraxia 1 (AOA1), AR	Date ym. 2001
	SETX	23064	Chr9: 134.13-134.22	Chr9: 55.25-55.32	Ataxia with oculomotor apraxia 2 (AOA2), AR	Moreira ym. 2004
	ATM	472	Chr11: 107.50-107.75	Chr5: 27.19-27.30	Ataxia telangiectasia (AT), AR	Savitsky ym. 1995
	MRE11A	4361	Chr11: 93.79-93.87	Chr21: 9.45-9.53	Ataxia-telangiectasia-like disorder (ATLD), AR	Stewart ym. 1999
	ATCAY	85300	Chr19: 3.8-3.87	Chr20: 58.68-58.70	Cayman Ataxia, AR	Bomar ym. 2003
	SACS	26278	Chr13: 22.80-22.88	Chr25: 18.24-18.28	Charlevoix-Saguenay spastic ataxia (ARSACS), AR	Engert ym. 2000
	FRDA	2395	Chr9: 70.84-70.88	Chr1: 91.36-91.37	Friedreich ataxia (FRDA), AR	Campuzano ym. 1996
	C10Orf2	56652	Chr10: 102.74-102.74	Chr28: 16.678-16.68	Infantile-onset spinocerebellar ataxia (IOSCA), AR	Nikali ym. 2005
	SIL1	64374	Chr5: 138.31-138.56	Chr11: 29.49-29.71	Marinesco-Sjögren syndrome (MSS), AR	Anttonen ym. 2005
	POLG	5428	Chr15: 87.66-87.68	Chr3: 55.22-55.24	Mitochondrial recessive ataxia syndrome (MIRAS), AR	Van Goethem ym. 2004
	SYNE1	23345	Chr6: 152.48-152.70	Chr1: 45.46-45.76	Spinocerebellar ataxia autosomal recessive 8 (SCAR8), AR	Gros-Louis et al. 2007
	SPTBN2	6712	Chro11: 66.21-66.25	Chr18: 53.66-53.70	Spinocerebellar ataxia 5 (SCA5), AD	Ikeda ym. 2006
	CACNA1A	773	Chr19: 13.22-13.25	Chr20: 51.82-52.04	Spinocerebellar ataxia 6 (SCA6), AD	Zhuchenko ym. 1997
	PRKCG	5582	Chr19: 59.07-59.10	Chr1: 106.38-106.39	Spinocerebellar ataxia 14 (SCA14), AD	Chen ym. 2003
	ITPR1	3708	Chr3: 4.51-4.86	Chr20: 15.75-16.07	Spinocerebellar ataxia 15 (SCA15), AD	van de Leemput ym. 2007
**Mouse phenotype**	*Camk4*	12326	Chr18: 33.08-33.31	Chr3: 4.07-4.26	Camk4$^{-/-}$ mouse, AR	Ribar et al. 2000
	Grid2	14804	Chr6: 63.19-64.60	Chr32: 18.29-19.72	Lurcher (*+/Lc*), AD; Hotfoot (*ho/ho*), AR	Zuo et al. 1997; Lalouette et al. 1998
	Scyl1	78891	Chr19: 5.76-5.77	Chr18: 54.69-54.71	Muscle deficient (*mdf/mdf*), AR	Schmidt et al. 2007
	Agtpbp1	67269	Chr13: 59.46-59.57	Chr1: 76.94-77.09	Purkinje cell degeneration (*pcd)* mice, AR	Fernandez-Gonzalez et al. 2002
	Reln	19699	Chr5: 21.40-21.86	Chr18: 19.27-19.76	Reeler (*rl/rl*), AR	D'Arcangelo et al. 1995
	Psap	19156	Chr10: 59.67-59.70	Chr4: 25.78-25.80	Saposin D$^{-/-}$ mouse, AR	Matsuda et al. 2004
	Rora	19883	Chr9: 68.45-69.18	Chr30: 28.32-28.35	Staggerer (*sg/sg*), AR	Hamilton et al. 1996
	Aars	234734	Chr8: 113.92-113.94	Chr5: 79.29-79.31	Sticky (*sti/sti*), AR	Lee ym. 2006
	Kcnj6	16522	Chr16: 94.86-95.11	Chr31: 34.88-35.13	Weaver (*wv/wv*), AR	Patil et al. 1995

AR=Autosomal recessive, AD=Autosomal dominant
