# Supplementary material for: A SEL1L Mutation Links a Canine Progressive Early-Onset Cerebellar Ataxia to the Endoplasmic Reticulum–Associated Protein Degradation (ERAD) Machinery
Source: PLoS Genet. 2012 Jun 14;8(6):e1002759. doi: 10.1371/journal.pgen.1002759 (PMC3375262; doi:10.1371/journal.pgen.1002759)
Supplement: Table S2 — Variants identified in candidate gene sequencing. (PDF) [file pgen.1002759.s002.pdf]

Table S2. Variants identified in candidate gene sequencing.

Gene (mRNA reference)	PCR amplicon	Variant	Position	Amino acid change	Segregation[1]
**SEL1L**	SEL1L_exo2	c.71-121T>C	Intron 1		yes
(XM_537530.2)	SEL1L_exo3	c.340+120A>T	Intron 3		yes
	SEL1L_exo3	c.340+133G>A	Intron 3		yes
	SEL1L_exo3	c.340+145T>G	Intron 3		yes
	SEL1L_exo4	c.508+43T>A	Intron 4		yes
	SEL1L_exo6	**c.669A>G**	**Exon 6**	**p. Glu223Glu > silent**	yes
	SEL1L_exo7-8	c.778-134C>T	Intron 6		yes
	SEL1L_exo7-8	c.778-98_99insGT	Intron 6		yes
	SEL1L_exo7-8	c.831+26_27insACTACCA	Intron 7		yes
	SEL1L_exo7-8	c.891+20T>C	Intron 8		yes
	SEL1L_exo10	c.1128+15delT	Intron 10		yes
	SEL1L_exo10	c.1128+111C>T	Intron 10		yes
	SEL1L_exo10	c.1128+155T>C	Intron 10		yes
	SEL1L_exo12	c.1254+75C>T	Intron 12		yes
	SEL1L_exo13	c.1255-243_244insGAGTATA	Intron 12		yes
	SEL1L_exo13	c.1255-63G>C	Intron 12		yes
	SEL1L_exo14	c.1333-43_44insATTT	Intron 13		yes
	SEL1L_exo16	c.1484-68C>T	Intron 15		yes
	SEL1L_exo18	**c.1869T>G**	**Exon 18**	**p. Ser623Ser > silent**	yes
	SEL1L_exo19	**c.1972T>C**	**Exon 19**	**p.Ser658Pro > missense**	yes
	SEL1L_exo20	c.2047-151T>C	Intron 19		yes
	SEL1L_exo20	c.2047-101_102insTAT	Intron 19		yes
	SEL1L_exo21a	**c.2364G>A**	**Exon 21**	**p. Pro788Pro > silent**	yes
**CEP128**	CEP128_exo7	c.572+35A>T	Intron 7		yes
(XM_547936.3)	CEP128_exo23	c.2970-15_18delGTTT	Intron 22		no
**GTF2A1**	GTF2A1_exo1	c.-100A>G	5'UTR		no
(XM_849814.1)	GTF2A1_exo1	c.-91G>A	5'UTR		no
	GTF2A1_exo4	c.401-7A>T	Intron 3		no
**STON2**	STON2_exo1	c.-106C>T	5' UTR		no
(XM_547937.3)	STON2_exo4c	**c.1392A>G**	**Exon 4**	**p.Glu464Glu > silent**	yes
	STON2_exo4f	c.2410+56A>G	Intron 4		yes
	STON2_exo5b	c.2730+108A>G	Intron 5		yes
	STON2_exo6	c.2731-40C>T	Intron 5		yes
**TSHR** (NM_001003285.1)	TSHR_exo_10b	c.1600C>A	**Exon 10**	**p.Arg534Ser > missense**	no*

[1]Segregation of variant with the phenotype in two cases and in two obligate carriers.

* Was homozygous in all four Finnish Hounds.
