# Supplementary material for: A SEL1L Mutation Links a Canine Progressive Early-Onset Cerebellar Ataxia to the Endoplasmic Reticulum–Associated Protein Degradation (ERAD) Machinery
Source: PLoS Genet. 2012 Jun 14;8(6):e1002759. doi: 10.1371/journal.pgen.1002759 (PMC3375262; doi:10.1371/journal.pgen.1002759)
Supplement: Table S3 — Control dogs from other breeds. (PDF) [file pgen.1002759.s003.pdf]

Table S3. Control dogs from other breeds.

Breed	n
Norwegian Elkhound	32
Alaskan Malamute	29
German Shepherd Dog	27
Lagotto Romagnolo	27
Havanese	26
Beagle	25
Jack Russell Terrier	25
Saluki	25
Irish Setter	23
Boxer	22
Russian Hound (Russkaja Gonzaja)	10
Australian Shepherd	2
Beauceron	2
Belgian Shepherd, Tervuren	2
Berger Picard	2
Bernese Mountain Dog	2
Black Russian Terrier	2
Bolognese	2
Border Collie	2
Border Terrier	2
Briard	2
Cairn Terrier	2
Chihuahua	2
Coton de tulear	2
Dachshund (long-haired)	2
English Springer Spaniel	2
Finnish Lapphund	2
Finnish Spitz	2
Flat-Coated Retriever	2
French Bulldog	2
Greyhound	2
Jagdterrier	2
Karelian Bear Dog	2
Kromfohrländer	2
Lhasa apso	2
Newfoundland	2
Nova Scotia Duck-Tolling Retriever	2
Papillon	2
Portuguese Podengo (wire coat)	2
Pyrenean Shepherd (rough coat)	2
Samoyed	2
Schipperke	2
Shih tzu	2
Siberian Husky	2
Skye Terrier	2
Smooth Collie	2
Swedish Vallhund	2
Tibetan Spaniel	2
Welsh Terrier	2
Akita Inu	1
Caucasian Shepherd Dog	1
**51 breeds**	**349**
